# Dietary patterns and fertility status in men: Mediterranean diet does make a difference in ameliorating the rise in male infertility problems due to changing lifestyle

**DOI:** 10.1097/JS9.0000000000000158

**Published:** 2023-03-06

**Authors:** Nirmala Sehrawat, Ujjawal Sharma, Mukesh Yadav, Varruchi Sharma, Abhijit Dey, Talha B. Emran, Anil K. Sharma, Kuldeep Dhama

**Affiliations:** a Department of Human Genetics and Molecular Medicine, School of Health Sciences, Central University of Punjab, Bathinda, Punjab, India, Department of Biotechnology, MMEC, Maharishi Markandeshwar (Deemed to be University), Mullana-Ambala, Haryana; b Department of Biotechnology, Sri Guru Gobind Singh College Sector 26, Chandigarh; c Department of Life Sciences, Presidency University, Kolkata, West Bengal; d Division of Pathology, ICAR-Indian Veterinary Research Institute, Izatnagar, Uttar Pradesh, India; e Department of Pharmacy, BGC Trust University Bangladesh, Chittagong, Bangladesh; f Department of Pharmacy, Faculty of Allied Health Sciences, Daffodil International University, Dhaka, Bangladesh

HIGHLIGHTS

- Infertility has been afflicting an increasing proportion of our society.
- Majority of the male infertility features are attributed to oligospermia.
- The present corresponding article highlights the salient predispose factors.
- One of the dietary models that adhere to the fundamentals of a pro-fertility diet is the MD.

Infertility has been afflicting an increasing proportion of our society, with an estimated more than 70 million couples of reproductive age encountering problems getting pregnant. Interestingly, half of them relate to male infertility issues in one way or the other^1^. Majority of the male infertility features are attributed to oligospermia (semen with low concentration of sperms), asthenozoospermia (nonmotility or decreased motility of spermatozoa), or teratozoospermia (spermatozoa with altered structure)^2^. The present corresponding article highlights the salient predispose factors responsible for causing male infertility problems, especially during the current era of western diets dominating the lifestyle, and the significant role of healthy dietary models with nutritional interventions such as Mediterranean diets (MDs) in enhancing fertility and ameliorating the problems related to male infertility. There has been an intricate and complex relationship between the qualitative and quantitative aspects of dietary nutrients and the quality of sperms, as evidenced through earlier studies where sperm energetic metabolism has been directly affected by not only the amount but the quality of nutrients as well. For example, a diet rich in saturated fats or a diet low in polyunsaturated fatty acids (PUFAs) having unbalanced omega-6/omega-3 PUFA negatively impact the sperm quality while supplementing the PUFA-rich diet ameliorates the above negative effects on sperm quality and preserving the male fertility^3^. It is suggested that a healthy diet based on plant foods or fish positively impacts sperm quality, increasing the mitochondrial enzyme activities responsible for gamete energetic metabolism and reducing oxidative damage, hence preventing male infertility.

Genetic and environmental factors are known to contribute toward male infertility. Among environmental factors environmental pollution, age, smoking, emotional stress, tight clothing, exposure to high temperatures, excessive alcohol consumption, exposure to electromagnetic radiation and pesticides, and a sedentary lifestyle significantly affect male fertility^1^. It is certain that some factors like environmental pollution, age, or radiation cannot be evaded^4^–^6^. Nevertheless, it has been observed that antioxidants, such as resveratrol, protect the sperm from oxidative stress and may act as one of the alternative therapeutics for male infertility^6^,^7^. Moreover, recent studies pointed toward the association of diet modulating semen quality^6^,^8^,^9^.

The ‘Western diet’ has emerged as the dominant dietary pattern in recent decades^3^. This diet is considered to be of high consumption of commercially processed foods, which are low in essential unsaturated fatty acids and dietary fiber but high in trans and saturated fats, simple carbohydrates, and animal proteins which are directly linked to augmenting oxidative stress that leads not only to metabolic disorders such as type 2 diabetes, obesity, and insulin resistance but also associated with a decline in fertility by decreasing the sperm quality^10^,^11^. Therefore, reactive oxygen species (ROS), proinflammatory cytokines, and aromatase activity, the enzyme that converts testosterone into estradiol, are all produced in greater amounts when white adipose tissue is present. However, type 2 diabetes, insulin resistance, and obesity in males increase the risk of secondary hypogonadism and reduced sex hormone-binding protein levels. Additionally, the process of fertilization and sperm motility are also negatively impacted by hyperglycemia^3^. The hallmarks of insulin resistance, hyperinsulinemia, and hyperglycemia, appear to be the cause of the decline in glucose uptake and metabolism in sperm^12^, possibly contributing to the impairment of sperm cells’ glycolysis which serves as the main source of energy for spermatozoa^13^. This theory is supported by the findings that insulin treatment reversed the decreased sperm motility observed in diabetic male rats^14^. Additionally, the chronic proinflammatory condition of the testicular microenvironment and/or excurrent ductal system, caused by an increase in ROS^15^, which is what causes the decline in sperm quality, is significantly influenced by leptin, a hormone released from fat cells in adipose tissue^15^,^16^.

Conversely, healthy dietary models are clearly connected with improved quality of sperms, implying that interventions based on nutrition may play an imperative role in the preservation of male fertility^3^. Male infertility prevention and/or treatment has also been shown to be quite successful with an appropriate intake of antioxidant molecules^17^. One of the dietary models that adhere to the fundamentals of a profertility diet is the MD. The MD is a manner of eating that is based on the classic recipes of Greece, Italy, and other Mediterranean Sea countries. Mediterranean food is popular in Italy, Greece, Turkey, Lebanon, Morocco, and a number of Middle Eastern and North African countries. As a result, no single MD exists. A significant portion of vegetables and fruit, whole grain goods, nuts, olive oil, and seafood are consumed frequently in MD; thus, this diet is labeled as rich in monounsaturated fatty acids, fiber, and antioxidants while low in saturated fatty acids. MD dietary model does bear similarities to the vegetarian diet (VD) model though VD lacks meat, meat products, seafood, etc. Some studies, though pointed out the negative impact of the VD model on semen quality^18^; however, it is still a matter of further investigation.

The MD has been linked to numerous health benefits, mostly because of its lipid-lowering, anti-inflammatory, and antioxidant properties^8^. In observational studies, consuming an MD has also been linked to greater semen quality. Numerous dietary natural polyphenols present in vegetables, fruits, and edible plants have been demonstrated to affect ROS homeostasis, mitochondrial metabolism, and biogenesis^19^,^20^. These plant bioactive compounds may have a significant contribution in the enhancement of male reproductive performance by modulating mitochondrial function and reducing oxidative stress^1^,^3^. The influence of fatty acids and cholesterol on testicular glutamyl aminopeptidase and gamma glutamyl transpeptidase activities, as well as favorable effects of a diet high in virgin olive oil, a key component of the MD, on male fertility, may be mediated in part by an increase in testicular sol dipeptidyl peptidase IV activity^21^. Foods high in flavonoids, which are also a part of the MD, have been linked to several biological processes that can protect male fertility^22^. Curcumin, which is found in the powdered rhizome of *Curcuma longa* used in the MD (turmeric latte and Mediterranean yellow rice), is a potent antioxidant that aids in the direct elimination of ROS and the indirect activation of antioxidant enzymes. Low-carbohydrate diets have been found to reduce sperm quality and damage testicular histology; therefore, curcumin supplements may improve the impaired function of sperms and testis by reducing inflammation, oxidative stress, and apoptosis^23^. In men with low-quality sperm, antioxidant-rich diets may enhance sperm quality by reducing oxidative stress-induced sperm damage and increasing hormone production, spermatozoa concentration, motility, and morphology^24^. Men’s fertility and sperm quality are also improved by increasing consumption of monounsaturated and PUFA in foods such as whole grain cereals, fish, seafood, and low-fat dairy products^25^. The benefits of MD can be strengthened further by increasing physical activity as part of a well-balanced healthy lifestyle.

The role of simple and complex carbohydrates in diet affecting sperm function is yet to be established. However, some evidence clearly suggest the negative impact of excess simple carbohydrates on sperm quality. Moreover, the protein-deficient diet or diet lacking specific amino acids does also form a strong basis as a potential risk for male infertility. The main cause of male infertility is oxidative stress; therefore, the proper synthesis, function, and vitality of spermatozoa depend on the balance between ROS and antioxidants. Molecules of life like fats, carbohydrates, and proteins are known to affect sperm quality as they presumably act upon the oxidative stress and testosterone levels through mitochondria which are the powerhouse for energy production, mediating ROS homeostasis, and hormone biosynthesis, thus affecting male fertility, while the effect is reversed by PUFA and polyphenol-rich diet. It would be beneficial to conduct future clinical trials with large sample sizes that focus on combining several antioxidants with taking advantage of their combined mechanisms of action for promoting ameliorating male fertility. People who follow the MD or any other anti-inflammatory diet may occasionally eat significantly more or less fruit, vegetable, or dairy items than other people. Despite the use of dietary indices to identify and enhance anti-inflammatory diet adherence, there is a lack of agreement over the application of certain MD methods across various nations and areas. Because there is uncertainty surrounding the definition of anti-inflammatory diets, there is a lot of variety in how they are operationalized. In order to produce high-quality nutrition intervention studies, future research should concentrate on resolving the drawbacks. An overview of the influence of the MD in improving male fertility and ameliorating infertility problems in men is depicted in Figure 1.

**Figure 1:**
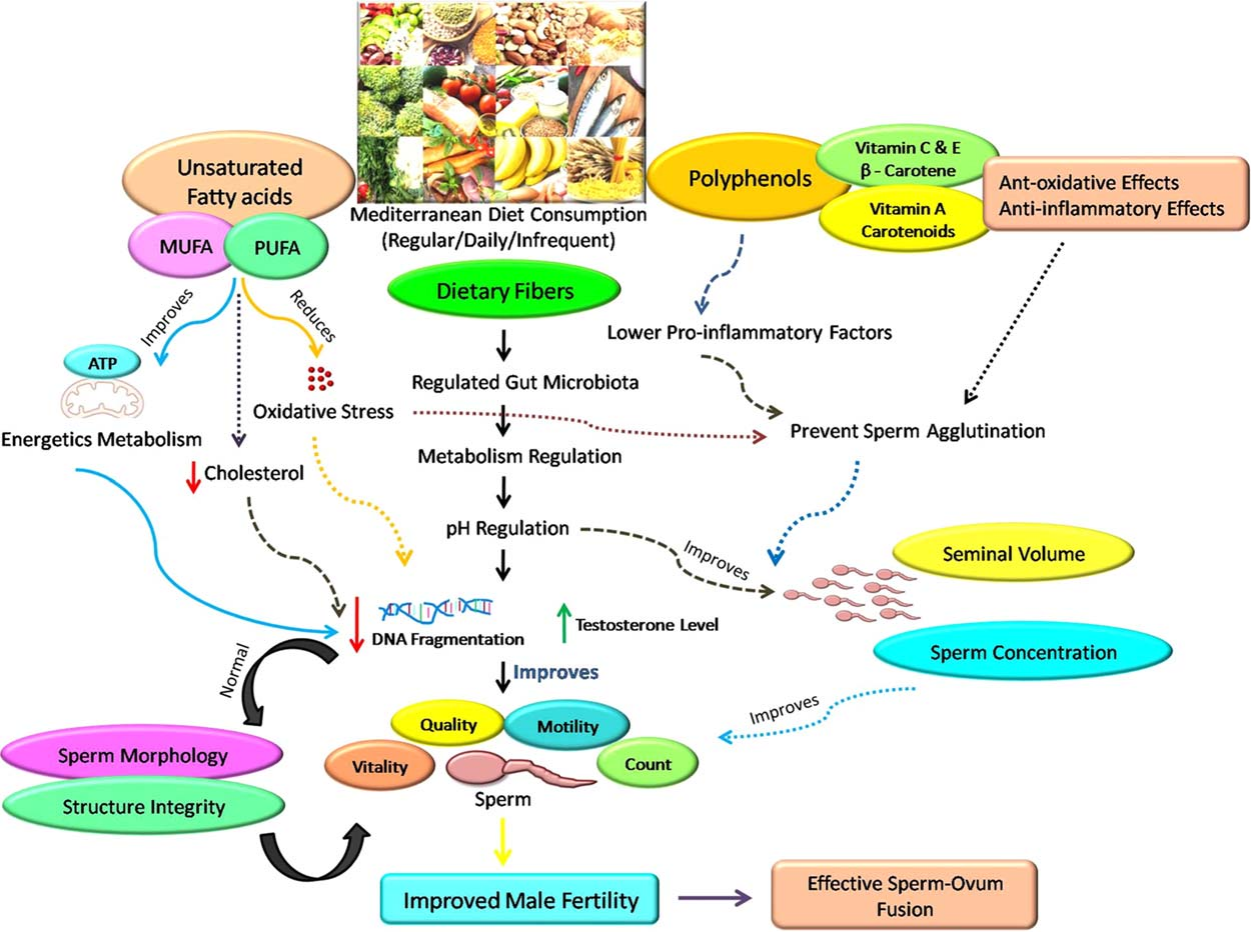
Dietary influence on male infertility. The dietary fibers enriched Mediterranean diet through gut microbiota homeostasis, bringing about the regulation of metabolism as well as pH, further resulting in an increase in seminal volume and sperm concentration leading to an overall improvement in male infertility. Moreover, unsaturated fatty acids [polyunsaturated fatty acids (PUFA) and monounsaturated fatty acids (MUFA)] could reduce oxidative stress and cholesterol, followed by lowering DNA fragmentation, again leading to improved sperm vitality, count, motility, and quality as well. Similarly, polyphenols exert antioxidative effects resulting in the lowering of proinflammatory factors and preventing sperm agglutination, hence leading to improved male infertility.

There seems to be a major influence of the dietary regimen on sperm quality, especially the MD, but still, there are a variety of other lifestyle risk factors as well, such as excess alcoholism, smoking, drug addiction, obesity, psychological stresses, overuse of caffeinated drinks, and sedentary lifestyle with lack of physical exercises^26^. Moreover, there is a strong possibility of biomagnification of the underlying negative consequences in subsequent generations. In order to mitigate and ameliorate the negative impacts, one has to extensively work on dietary and other lifestyle modifications which could further improve the male reproductive potential. Though there is a strong need to delineate the individual risk factors involved and the sequence of events as an outcome of multiple factors involved simultaneously. However, the role of diet is certainly at center stage in the current context, as male infertility cases are constantly on the rise. Maintaining a positive dietary and healthy lifestyle, along with early counseling and appropriate clinical interventions, do hold the key to cope up with the impact of risk factors influencing sperm quality and male reproductive health. Given the relevance of the role that diet plays in male infertility, which is occurring at an exponentially increasing rate today, additional studies concentrating on the underlying molecular mechanisms of action of natural substances and nutrients are essential to formulate innovative dietary approaches to preserve and enhance male reproductive potential and treat male fertility problems.

## Ethical approval

Not applicable.

## Sources of funding

No funding was received.

## Author contribution

N.S.: conceptualization, data curation, and writing – original draft preparation, reviewing, and editing. U.S., V.S., A.D., A.K.S., and K.D.: data curation and writing – original draft preparation, reviewing, and editing. T.B.E.: writing – reviewing and editing, visualization, and supervision.

## Conflicts of interest disclosure

The authors declare that they have no financial conflict of interest with regard to the content of this report.

## Research registration unique identifying number (UIN)

None.

## Guarantor

Talha Bin Emran, PhD, Associate Professor, Department of Pharmacy, BGC Trust University Bangladesh, Chittagong 4381, Bangladesh. Tel: +88 030 335 6193, fax: +88 031 255 0224. https://orcid.org/0000-0003-3188-2272.

## Data statement

The data in this correspondence article is not sensitive in nature and is accessible in the public domain. The data is therefore available and not of a confidential nature.

## Provenance and peer review

Not commissioned, internally peer-reviewed.
